# Production of viable seeds from the seedling lethal mutant *ppi2-2* lacking the atToc159 chloroplast protein import receptor using plastic containers, and characterization of the homozygous mutant progeny

**DOI:** 10.3389/fpls.2014.00243

**Published:** 2014-06-04

**Authors:** Akari Tada, Fumi Adachi, Tomohiro Kakizaki, Takehito Inaba

**Affiliations:** ^1^Department of Agricultural and Environmental Sciences, Faculty of Agriculture, University of MiyazakiMiyazaki, Japan; ^2^National Institute of Vegetable and Tea ScienceTsu, Japan

**Keywords:** albino, *Arabidopsis*, chloroplast, *ppi2-2* mutant, protein import, seed

## Abstract

Biogenesis of chloroplasts is essential for plant growth and development. A number of homozygous mutants lacking a chloroplast protein exhibit an albino phenotype. In general, it is challenging to grow albino *Arabidopsis* plants on soil until they set seeds. Homozygous albino mutants are usually obtained as progenies of heterozygous parents. Here, we describe a method of recovering seeds from the seedling lethal *Arabidopsis* mutant *ppi2-2*, which lacks the atToc159 protein import receptor at the outer envelope membrane of chloroplast. Using plastic containers, we were able to grow homozygous *ppi2-2* plants until these set seed. Although the germination rate of the harvested seeds was relatively low, it was still sufficient to allow us to further analyze the *ppi2-2* progeny. Using *ppi2-2* homozygous seeds, we were able to analyze the role of plastid protein import in the light-regulated induction of nuclear genes. We propose that this method be applied to other seedling lethal *Arabidopsis* mutants to obtain homozygous seeds, helping us further investigate the roles of plastid proteins in plant growth and development.

## INTRODUCTION

Plastids such as chloroplasts in photosynthetic plant cells are believed to have evolved from a cyanobacterium-like ancestor (Dyall et al., 2004). During evolution, most of the genes encoded by the bacterial ancestor were transferred to the nuclear genome of the host. Therefore, the expression of nuclear genes encoding plastid proteins and the import of those proteins into plastids are essential for plastid biogenesis. The key player involved in delivering nuclear-encoded proteins into plastids is the translocon at the outer envelope membrane of chloroplasts (TOC) and the translocon at the inner envelope membrane of chloroplasts (TIC) complex (Inaba and Schnell, 2008; Li and Chiu, 2010; Jarvis and Lopez-Juez, 2013). The TOC–TIC complex was first isolated through biochemical purification (Kessler et al., 1994; Schnell et al., 1994). Molecular genetic analysis of identified components using *Arabidopsis* indicated that these were indeed indispensable for plastid biogenesis (Jarvis et al., 1998; Bauer et al., 2000; Chou et al., 2003; Constan et al., 2004a,b; Ivanova et al., 2004; Kubis et al., 2004; Inaba et al., 2005; Kovacheva et al., 2005; Teng et al., 2006; Kikuchi et al., 2013).

Because of their key roles in plastid protein import, a number of mutants defective in TOC or TIC proteins exhibit severe developmental arrest, resulting in embryo and seedling lethality. These lethal phenotypes have made it difficult to characterize in more detail the roles of the TOC–TIC complex in plant growth and development. For instance, the homozygous *plastid protein import 2* (*ppi2*) mutant that lacks the major protein import receptor of plastids, atToc159, exhibits seedling lethality due to its severe albino phenotype (Bauer et al., 2000; Kakizaki et al., 2009). Therefore, we can only obtain bulk seeds from heterozygous *ppi2* (*ppi2/+*) plants. When the progeny of *ppi2-2/+* is grown in the dark, it is virtually impossible to discriminate between homozygous *ppi2-2* and the heterozygous *ppi2-2/+*. Hence, to further uncover the role of plastid protein import in plant growth and development, it is necessary to propagate seeds from seedling lethal, albino mutants such as the homozygous *ppi2*.

In this paper, we describe a method for generating viable seeds from the seedling lethal *Arabidopsis* mutant *ppi2-2*, which lacks the major protein import receptor of plastids (Bauer et al., 2000; Kakizaki et al., 2009). Using these seeds, we investigated the photomorphogenic response of the *ppi2-2* mutant and showed that the TOC–TIC pathway and the light-induced gene expression are tightly coordinated with each other. Our method also provides clues on how to obtain viable seeds from other albino *Arabidopsis* plants, allowing us to uncover the roles of plastid proteins in plant growth and development in more detail.

## MATERIALS AND METHODS

### PLANT MATERIALS

All experiments were performed on *Arabidopsis thaliana* accession Columbia (Col-0). The *ppi2-2* mutant has been described elsewhere (Kakizaki et al., 2009). Wild-type and *ppi2-2/+* seeds were obtained from plants grown on soil.

### GROWTH CONDITIONS FOR RECOVERING HOMOZYGOUS *ppi*2-2 SEEDS

**FIGURE 1:**
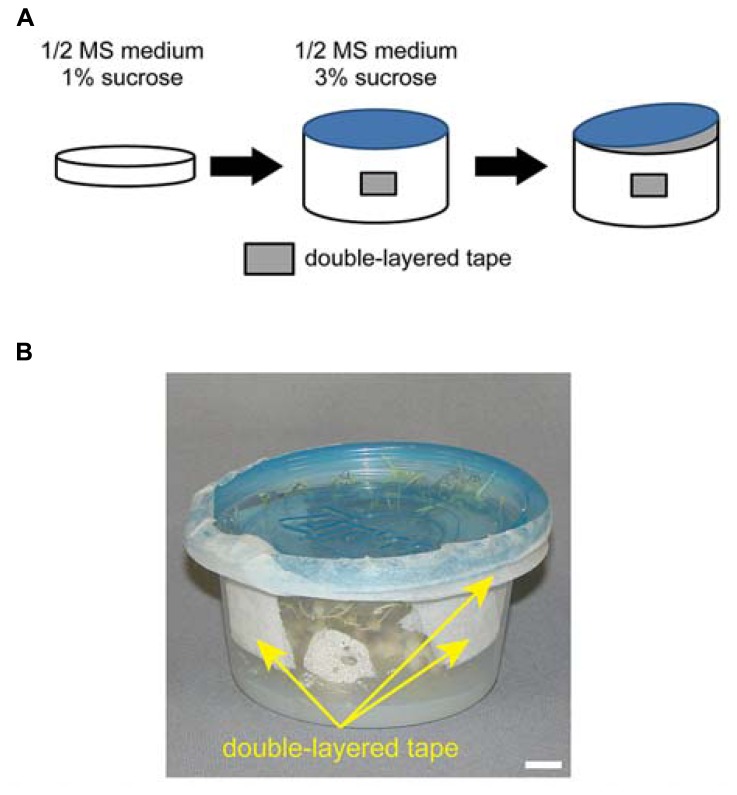
**Overview of homozygous *ppi2-2* cultivation using plates and Ziploc^®^ containers. (A)** Homozygous *ppi2-2* plants were first grown in MS plates (left) and then transferred into a Ziploc^®^ container (middle). The Ziploc^®^ container has four holes covered with double-layered surgical tape. At a later stage, the lid of the Ziploc^®^ container was partially opened (right), and a gap between the lid and the container was sealed with double-layered surgical tape. **(B)** Ziploc^®^ container used in this study. Bar = approximately 1 cm.

**FIGURE 2:**
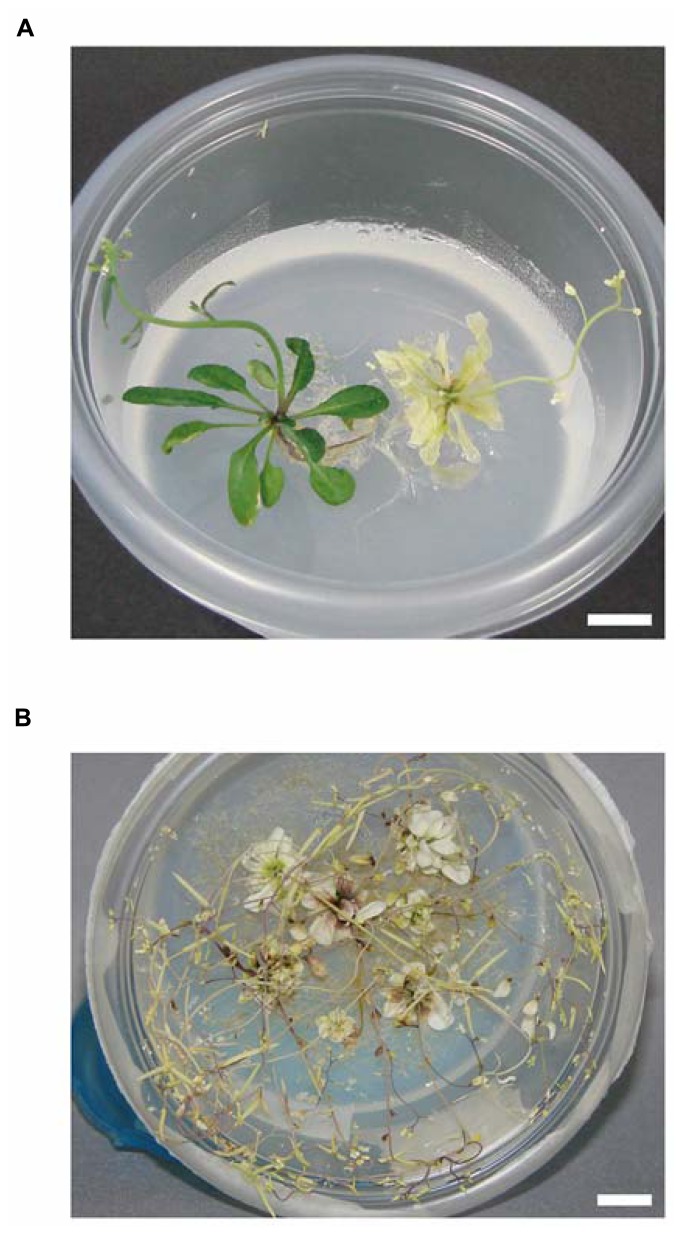
**Phenotype of homozygous *ppi2-2* plants grown in Ziploc^®^ containers. (A)** Representative phenotype of wild-type (left) and *ppi2-2* (right) plants grown in Ziploc^®^ containers. After transferring the plants from plates, they were grown in a Ziploc^®^ container for 10 days. Bar = approximately 1 cm. **(B)** At a later stage, the *ppi2-2* plants set seeds in the Ziploc^®^ container. Bar = approximately 1 cm.

An overview of the growth method is summarized in **Figure 1A**. The progeny of *ppi2-2/+* plants were first grown on plates (150 mm in diameter) containing 0.5% agar, 1% sucrose, and 0.5× MS salts at pH 5.8. To synchronize germination, all seeds were maintained at 4°C for 2 days after sowing. Plants were grown under continuous white light (80 μmol m^-^^2^ s^-^^1^, unless specified) at 22°C and 50% relative humidity in a growth chamber (LPH-350S, NK system). After 14–18 days, homozygous *ppi2-2* plants were transferred to small size, round-shaped Ziploc^®^ containers (width 108 mm × depth 108 mm × height 56 mm, 236 ml container size, Asahi Kasei Co. Ltd., Japan; see **Figures 1B** and **2**) containing 0.8% agar, 3% sucrose, and 0.5× MS salts at pH 5.8. We believe this container is most similar to the Ziploc^®^ brand Container with the Smart Snap^®^ Seal Extra Small Bowl (8 ounces, S.C. Johnson & Son, Inc., Howe St Racine, WI, USA) in the United State. Typically, 14–18 days old *ppi2-2* plants have four to six small true leaves. It is important to choose well-developed *ppi2-2* plants for subsequent cultivation in Ziploc^®^ containers. To avoid excess humidity and facilitate air circulation in the pot, each pot had four holes that were sealed with two layers of surgical tape (**Figure 1**). We made those holes using a knife. In most cases, we placed five to seven plants in each pot. At this point, the lid was tightly sealed and taped with surgical tape (**Figure 1A**, middle). We continued to grow the plants until they started bolting (**Figure 2A**, right). Once the plants had started bolting, the lid was partially opened and taped with two layers of surgical tape (**Figure 1B**). The plants were then harvested after they set seeds (**Figure 2B**). The harvested plants were dried in envelopes under laboratory condition for at least 2 weeks and then the seeds were collected.

### IMPORTANT NOTES

(1) The reason we use Ziploc^®^ containers instead of typical tissue culture pots is because Ziploc^®^ container is inexpensive and autoclavable.
(2) It is critical to obtain healthy *ppi2-2* plants from plates and use these for subsequent cultivation in Ziploc^®^ containers. Generally, if the *ppi2-2* plants are too old or subjected to stress (e.g., accumulation of anthocyanin) on the plates, the rate of success in obtaining seeds is significantly lower.
(3) Once the lid of the container is opened, it is important to prevent the development of excess humidity in the growth chamber. If the humidity is too high, the flowers of the *ppi2-2* plants will wilt and fail to produce seeds. We usually grow plants at 50% relative humidity until they set seeds.
(4) As described elsewhere (Shipman-Roston et al., 2010), it is critical to provide 3% sucrose to support the heterotrophic growth of albino plants. Because high sucrose content also facilitates senescence, we supplied 3% sucrose only in the Ziploc^®^ containers.

### GROWTH CONDITIONS FOR LIGHT EXPOSURE EXPERIMENTS

Plants were grown on 0.5% agar medium containing 1% sucrose and 0.5× MS salts at pH 5.8. To synchronize germination, all seeds were maintained in the dark at 4°C for 3 days after sowing. After low temperature treatment, seeds were exposed to white light for 8 h at 22°C and then returned to the dark for 4 days. Dark-grown plants were harvested and frozen in liquid nitrogen under a dim green light. A fraction of the dark-grown plants was then exposed to continuous white light for 24 h. After exposure to continuous white light, the plants were harvested and ground in liquid nitrogen for subsequent analysis.

### RNA ISOLATION AND REAL-TIME PCR ANALYSIS

Total RNA was extracted from aerial tissues of wild-type and mutant plants using an RNAiso plus reagent (Takara) as described elsewhere (Kakizaki et al., 2009). We prepared three independent RNA samples for each treatment. Each RNA sample was prepared from ~30 plants (equivalent to three spots in **Figure 4A**). cDNA was then synthesized using the PrimeScript^TM^ RT reagent kit (Takara) using a random hexamer and oligo d(T) primers. Real-time PCR was performed on a Thermal Cycler Dice Real-Time System (Takara) using SYBR Premix Ex Taq II (Takara) as previously described (Kakizaki et al., 2009). The primers used for real-time PCR are listed in **Table 1**. The transcript level of each gene was normalized to that of *ACTIN2*.

**Table 1:** List of gene-specific primers used in real-time PCR analysis.

Gene	AGI code	Forward primer	Reverse primer
*ACTIN2*	At3g18780	5′-GCACCCTGTTCTTCTTACCG-3′	5′-AACCCTCGTAGATTGGCACA-3′
*PsaF*	At1g31330	5′-CTGAATCTGCCCCTGCTCTT-3′	5′-AACCGTCTGACCCGCATAAC-3′
*PsbO1*	At5g66570	5′-AACGGCTAACCAGTGCCCTA-3′	5′-CTGGAGGAGCGTTCTTGCTT-3′
*SSU1A*	At1g67090	5′-CCTCAAAACTTTATCCCCCATC-3′	5′-AATATGTCTCGCAAACCGGAAA-3′
*LHCB3.1*	At5g54270	5′-TGAACATAACCTTTCTTGTTCCTC-3′	5′-GAGCATTGTAGATTTAGCTGTGAGA-3′
*PDH-E1α*	At1g01090	5′-TGCAAAGGAAGCAGAGCTAAAG-3′	5′-CCTCACATCTGTACCGTCCATC-3′
*UBC*	At5g25760	5′-CATCCTGAGCCGGACAGTCC-3′	5′-TAGCGGCGAGGCGTGTATAC-3′

## RESULTS

### CHARACTERIZATION OF SEEDS HARVESTED FROM HOMOZYGOUS *ppi*2-2 MUTANTS

We harvested the seeds from the 10 Ziploc^®^ containers (~50 plants). The yield of seeds in each experiment depended on the condition of the *ppi2-2* plants in the container. After four independent experiments, we obtained 0.26 g of homozygous *ppi2-2* seeds.

**FIGURE 3:**
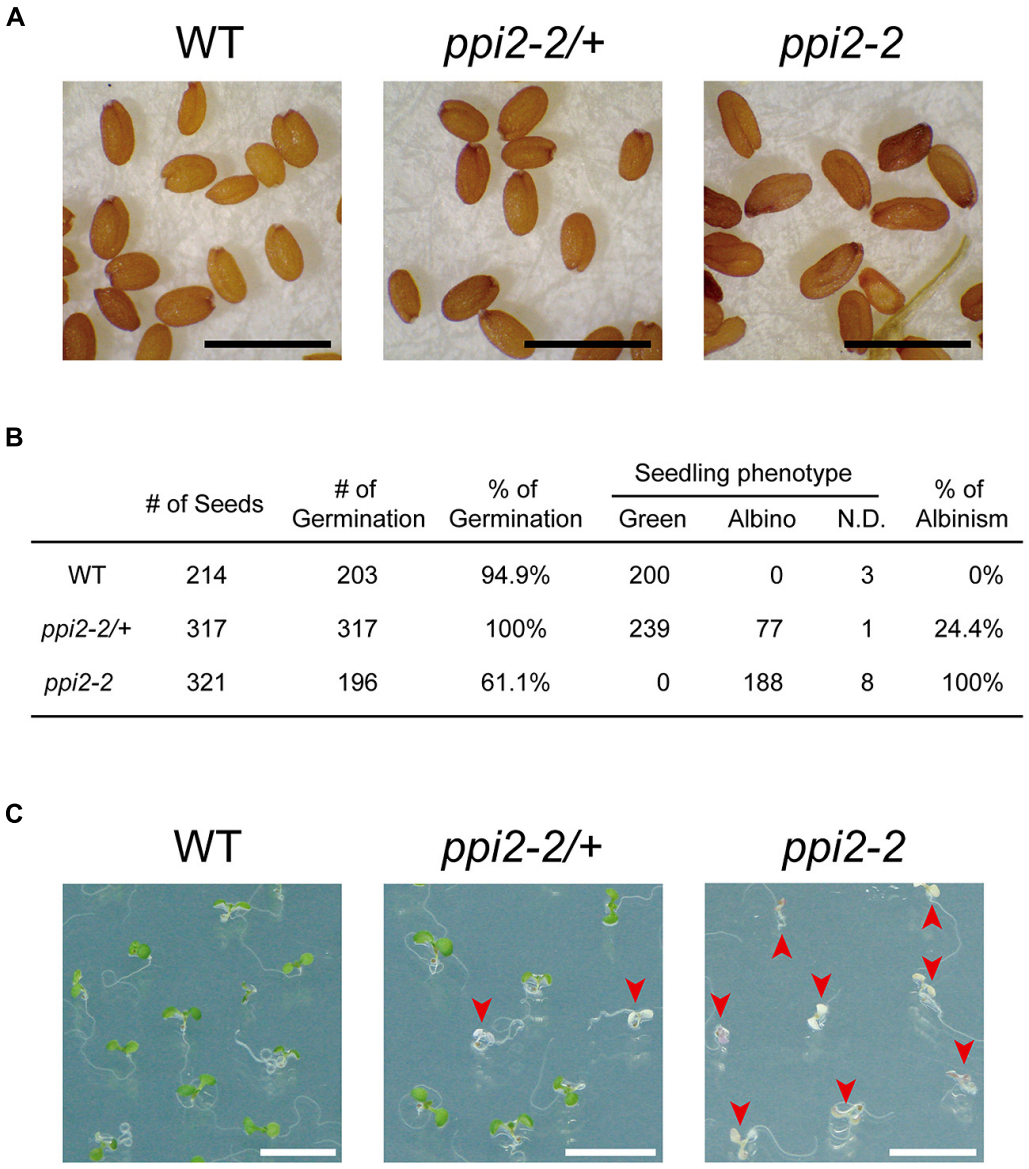
**Analysis of seeds derived from wild-type, heterozygous *ppi2-2*, and homozygous *ppi2-2* plants. (A)** The appearance of dry seeds derived from wild-type (WT), heterozygous *ppi2-2* (*ppi2-2/+*), and homozygous *ppi2-2* (*ppi2-2*) plants. Black bars = approximately 1 mm. **(B)** Analysis of germination rate and seedling phenotypes. Phenotypes were determined 6 days after the transfer of the plates to a growth chamber. Some of those seedlings could not be designated as green or albino due to growth retardation and thus were classified as “not determined (N.D.).” **(C)** Phenotype of seedlings germinated from wild-type, *ppi2-2/+*, and *ppi2-2* seeds. Arrowheads indicate albino plants. White bars = approximately 1 cm.

To determine whether the harvested seeds could be used for further analysis, we next examined the seeds harvested from wild-type, heterozygous *ppi2-2* (*ppi2-2/+*), and *ppi2-2* plants by microscopy. As shown in **Figure 3A**, the *ppi2-2* seeds were elongated in shape compared to those of the wild-type and *ppi2-2/+* seeds. Nonetheless, most of these elongated seeds did not look like aborted seeds.

When the wild-type, *ppi2-2/+*, and *ppi2-2* seeds were sown on MS plates, at least 90% of the wild-type and *ppi2-2/+* seeds germinated (**Figure 3B**). In contrast, only 60% of the *ppi2-2* seeds germinated 6 days after their transfer to the growth chamber (**Figure 3B**). The reason *ppi2-2* seeds exhibited a low germination rate remains unclear. Seed development is divided into two major phases, designated as the embryo and endosperm development phase and the seed maturation phase (West and Harada, 1993). Our previous observation suggested that embryo development of *ppi2-2* seeds was normal (Kakizaki et al., 2009). Consistent with this observation, the germination rate of *ppi2-2* seeds harvested from the *ppi2-2/+* plants was normal (**Figure 3B**, 25% of the *ppi2-2/+* progeny was *ppi2-2*). Hence, a possible explanation for the low germination rate of *ppi2-2* seeds is insufficient maturation due to high humidity in the pots. It is also possible that the growth retardation of *ppi2-2* plants affects the seed development on those plants.

We also investigated the phenotype of seedlings germinated from the wild-type, *ppi2-2/+*, and *ppi2-2* seeds. As shown in **Figures 3B,C**, all of the wild-type progeny exhibited a green phenotype, whereas ~25% of the *ppi2-2/+* progeny was albino. In contrast, 100% of the *ppi2-2* progeny exhibited an albino phenotype (**Figures 3B,C**).

In conclusion, we were able to recover viable seeds from homozygous *ppi2-2* seedlings. Furthermore, all of the *ppi2-2* progeny exhibited an albino phenotype. Although the germination rate of *ppi2-2* was relatively low, it was still sufficient to further analyze its progeny. We conclude that our method allows us to harvest seeds from the seedling lethal mutant *ppi2-2*.

### PHOTOMORPHOGENIC RESPONSE IN *ppi*2-2 MUTANTS: THE NEW METHOD HELPS US FURTHER UNDERSTAND THE ROLES OF PLASTID PROTEINS IN PLANT GROWTH AND DEVELOPMENT

**FIGURE 4:**
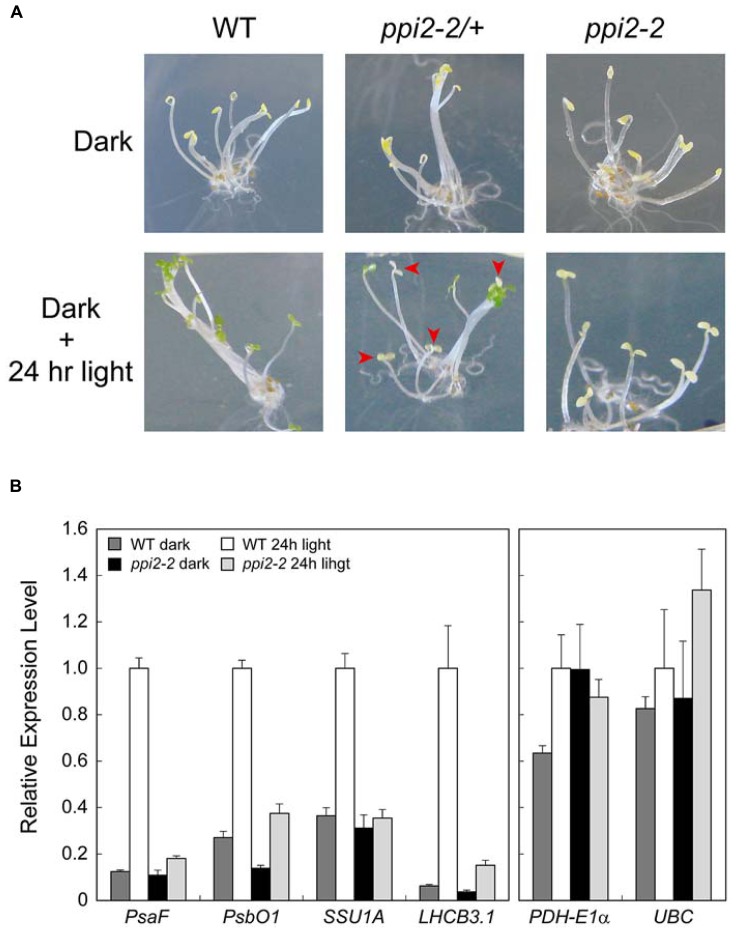
**The light response of seedlings germinated from wild-type (WT), heterozygous *ppi2-2* (*ppi2-2/+*), and homozygous *ppi2-2* (*ppi2-2*) seeds. (A)** Phenotype of wild-type, *ppi2-2/+*, and *ppi2-2* progenies grown in the dark (Dark). The plants were then exposed to continuous white light for 24 hr (Dark + 24 hr light). Arrowheads indicate homozygous *ppi2-2* plants found in the *ppi2-2/+* progeny. **(B)** Response of photosynthesis-related (left) and non-photosynthetic (right) genes upon light illumination. Expression levels were analyzed by real-time PCR and normalized to that of *ACTIN2*. The expression level in the light-illuminated wild-type (WT 24-h light) was set to 1. Each bar represents the mean of three independent samples. Error bars represent 1 SE.

Obtaining viable seeds from seedling lethal albino plants helps us uncover the roles of plastid proteins in plant growth and development in more detail. For instance, the role of plastid protein import in a photomorphogenic response remains unclear. This is because we cannot discriminate between *ppi2-2/+* and *ppi2-2* seedlings grown in the dark. When wild-type, *ppi2-2/+*, and *ppi2-2* seeds were germinated and grown in the dark for 4 days, all of the plants exhibited an etiolated phenotype regardless of their genotype (**Figure 4A**, upper panel). When wild-type plants were exposed to continuous white light for 24 h, their cotyledons turned green (**Figure 4A**, lower left panel). In contrast, *ppi2-2* plants opened their cotyledons after 24 h of light illumination, although they did not turn green (**Figure 4A**, lower right panel). The *ppi2-2/+* progeny turned green upon light illumination, whereas the segregated *ppi2-2* plants did not exhibit greening (**Figure 4A**, lower middle panel).

We next investigated the expression of photosynthesis-related genes upon light illumination in *ppi2-2* plants. For this analysis, we chose nuclear-encoded genes involved in photosynthetic electron transport (*PsaF*, *PsbO1*, and *LHCB3.1*) and CO_2_ fixation (*SSU1A*). In the wild-type, the expression of photosynthesis-related genes such as *PsaF*, *PsbO1*, *SSU1A*, and *LHCB3.1* was induced upon light illumination (**Figure 4B**, left panel). In contrast, light induction of these genes was compromised in the *ppi2-2* mutant (**Figure 4B**, left panel). We also confirmed that the expression of non-photosynthetic genes encoding pyruvate dehydrogenase E1α subunit (*PDH-E1*α) and ubiquitin conjugating enzyme (*UBC*). These genes have been shown to be expressed constitutively in *Arabidopsis* (Ivanova et al., 2004; Czechowski et al., 2005). As shown in **Figure 4B** (right panel), they did not show strong induction upon illumination. These data indicate that functional TOC machinery is a prerequisite for the rapid induction of photosynthesis-related genes upon light illumination. This also suggests that a tight coordination between plastid protein import and light-regulated gene expression would help prevent the accumulation of non-imported precursor proteins in the cytosol.

## CONCLUSION AND POSSIBLE APPLICATIONS

This report describes a method for obtaining viable seeds from the seedling lethal *ppi2-2* mutant using Ziploc® containers. Four independent experiments allowed us to obtain ~10,000 (0.26 g) homozygous *ppi2-2* seeds. We also confirmed that at least 60% of the harvested seeds were able to germinate (**Figure 3B**). Establishment of this new growth method allowed us to analyze the roles of plastid protein import in light-induced gene expression. Characterization of the homozygous *ppi2-2* progeny revealed that *ppi2-2* mutants failed to induce photosynthesis-related genes upon light illumination (**Figure 4**). This indicates that the integrity of the protein import apparatus plays a critical role in the rapid induction of photosynthesis-related genes upon light illumination. In contrast, the *ppi2-2* mutation did not affect cotyledon opening upon light illumination (**Figure 4A**). These results demonstrated that investigations involving the progeny of homozygous albino plants will help us further understand the role of plastid protein import in plant growth and development.

Although we used *ppi2-2* plants as the model in our experiments, we would like to propose that the method could be applied to other *Arabidopsis* mutants that are described as “seedling lethal.” **Table 2** shows a comprehensive list of seedling lethal *Arabidopsis* mutants lacking either an outer or inner envelope membrane protein in chloroplasts. Because many mutants that are defective in an envelope membrane protein exhibit an embryo lethal phenotype (Hormann et al., 2004; Baldwin et al., 2005; Inaba et al., 2005; Patel et al., 2008; Hsu et al., 2010), the number of albino mutants that can be used for investigations is limited. Instead, we found that the method can be extended to other albino mutants. It has been suggested that a number of seedling lethal *Arabidopsis* mutants are associated with chloroplast dysfunction (Budziszewski et al., 2001). An exhaustive analysis of *Arabidopsis* mutants lacking a chloroplast protein revealed that more than 50 mutants exhibited albino, pale green, and other chloroplast-associated phenotypes (Myouga et al., 2010). Hence, we anticipate that seeds from some of these albino/seedling lethal mutants can be obtained using our method.

**Table 2:** List of seedling lethal *Arabidopsis* mutants lacking an outer or inner envelope chloroplast membrane protein.

Mutant name	AGI code	Localization	Reference
*ppi2 (toc159)*	At4g02510	OM	Bauer et al. (2000), Kakizaki et al. (2009)
*tic20-I*	At1g04940	IM	Teng et al. (2006)
*cia5/pic1*	At2g15290	IM	Teng et al. (2006), Duy et al. (2007)
*plsp1*	At3g24590	Env/Thy	Inoue et al. (2005)
*apg1*	At3g63410	IM	Motohashi et al. (2003)
*tic100*	At5g22640	IM	Kikuchi et al. (2013)
*tic56*	At5g01590	IM	Kikuchi et al. (2013)
*mgd1*	At4g31780	IM	Kobayashi et al. (2007)
*vipp1*	At1g65260	IM/Thy	Zhang et al. (2012)

*OM, outer envelope membrane; IM, inner envelope membrane; Env, envelope membrane; Thy, thylakoid membrane.*

In summary, we have developed a method for recovering viable seeds from the seedling lethal *Arabidopsis* mutant *ppi2-2*. Our method can be applied to other albino *Arabidopsis* mutants, helping us further understand the roles of chloroplast proteins in plant growth and development.

## Conflict of Interest Statement

The authors declare that the research was conducted in the absence of any commercial or financial relationships that could be construed as a potential conflict of interest.
